# DNA/RNA Detection Using DNA-Templated Few-Atom Silver Nanoclusters

**DOI:** 10.3390/bios3020185

**Published:** 2013-04-23

**Authors:** Judy M. Obliosca, Cong Liu, Robert Austin Batson, Mark C. Babin, James H. Werner, Hsin-Chih Yeh

**Affiliations:** 1 Department of Biomedical Engineering, Cockrell School of Engineering, University of Texas at Austin, Austin, TX 78712, USA; E-Mails: jmobliosca@utexas.edu (J.M.O.); cong.liu@utexas.edu (C.L.); abatson@utexas.edu (R.A.B.); 2 College of Natural Sciences, University of Texas at Austin, Austin, TX 78712, USA; E-Mail: markcbabin@gmail.com; 3 Center for Integrated Nanotechnologies, Los Alamos National Laboratory, Los Alamos, NM 78745, USA

**Keywords:** DNA/RNA detection, few-atom silver nanoclusters, single-nucleotide polymorphism detection, turn-on sensors

## Abstract

DNA-templated few-atom silver nanoclusters (DNA/Ag NCs) are a new class of organic/inorganic composite nanomaterials whose fluorescence emission can be tuned throughout the visible and near-IR range by simply programming the template sequences. Compared to organic dyes, DNA/Ag NCs can be brighter and more photostable. Compared to quantum dots, DNA/Ag NCs are smaller, less prone to blinking on long timescales, and do not have a toxic core. The preparation of DNA/Ag NCs is simple and there is no need to remove excess precursors as these precursors are non-fluorescent. Our recent discovery of the fluorogenic and color switching properties of DNA/Ag NCs have led to the invention of new molecular probes, termed NanoCluster Beacons (NCBs), for DNA detection, with the capability to differentiate single-nucleotide polymorphisms by emission colors. NCBs are inexpensive, easy to prepare, and compatible with commercial DNA synthesizers. Many other groups have also explored and taken advantage of the environment sensitivities of DNA/Ag NCs in creating new tools for DNA/RNA detection and single-nucleotide polymorphism identification. In this review, we summarize the recent trends in the use of DNA/Ag NCs for developing DNA/RNA sensors.

## 1. Introduction

The detection and identification of specific nucleic acids (either DNA or RNA) is an enabling technology for a number of fields, including pathogen identification [1], the recognition of genetic mutations [2], and forensic analysis [3]. For many applications, quantification of the amount of nucleic acids present can be just as important as the identification of a given DNA sequence. For example, deletions of tumor-suppressor genes and copy number increases of oncogenes were found in many forms of cancer [4]. Other than measuring changes in gene copy number, quantitative detection of circulating DNA can also be a useful diagnostic tool for the progression of cancer [5]. By counting parental haplotypes in maternal plasma, it is now possible to non-invasively detect certain genomic diseases in an unborn fetus [6]. On a cellular level, methods to directly count the copy number of individual messenger RNA molecules in individual cells, such as single-molecule fluorescence *in situ* hybridization [7], have led to improved models of cellular gene regulation and new insights into the stochastic nature of gene expression [8,9]. 

A number of methods have been developed for the detection and quantification of specific DNA or RNA, such as real-time quantitative polymerase chain reaction (qPCR) [10], DNA sequencing [6,11], and DNA microarrays [12]. In DNA microarrays, surface-bound oligonucleotides (oligos) are used to capture specific nucleic acid targets present in solution (these targets are usually PCR pre-amplified). Surface immobilization of the captured oligos allows the sample to be vigorously washed to remove unbound fluorescent probes, facilitates readout in an imaging format, and enables highly multiplexed analysis, as different capture oligos are immobilized at different locations on the microarray surface. 

In contrast to DNA microarray analysis methods, specific DNA or RNA can also be directly detected in solution (often termed homogeneous detection). Unlike surface-immobilized assays, homogeneous detection in solution generally precludes separation or wash steps. Since unbound reporters are not washed away, homogeneous detection often relies on the use of probes or indicators that give a unique signature (e.g., fluorescence [10,13,14] or scattering [15]) in the presence of a specific nucleic acid target. Commonly used fluorescent probes for the homogeneous detection of DNA include Taqman probes [10,16] and molecular beacons (MBs) [13]. Both Taqman probes and MBs are oligonucleotides labeled with a fluorophore and a quencher, generating a donor-acceptor fluorescence resonance energy transfer (FRET) pair [17,18]. While these FRET-based methods have been successfully commercialized for the homogeneous detection of specific DNA, they have drawbacks. In particular, FRET probes are expensive since they require two moieties to be attached to the same DNA strand. In addition, FRET probes that contain either the fluorophore or the quencher (so-called singly-labeled impurities) contribute to a high fluorescence background or a reduced signal. Moreover, for MBs, a sophisticated thermodynamic analysis is needed for stem-loop sequence selection and the level of quenching (either Förster or contact quenching) in a FRET probe is never 100% efficient, yielding an immutable noise floor.

To overcome the limitations of these commercialized FRET probes, many groups are working on new homogenous sensing methods that do not rely on FRET to indicate the presence of a particular nucleic acid target. Some of these new methods use nanomaterials for sensing DNA hybridization, including gold nanoparticles [19,20,21] and carbon nanotubes [22,23]. A distinct advantage of optical nanomaterials over conventional organic dyes is their superior photostability, enabling long analysis times. DNA-templated silver nanoclusters (DNA/Ag NCs) [24,25,26,27,28,29,30,31], in particular, have recently emerged as a new class of fluorescent probes for the homogeneous detection of DNA, showing the capability to differentiate target sequences down to the single-nucleotide level [31,32,33]. Owing to their unique fluorogenic [29,30] and color-switching properties [30,31] (not before seen in organic dyes or semiconductor quantum dots), DNA/Ag NCs are expected to find more applications in analytical chemistry and quantitative biology in the years to come. In this review we present the latest progress of DNA/Ag NCs for DNA/RNA detection. 

## 2. DNA-Templated Silver Nanoclusters

Noble metal nanoclusters are collections of small numbers of gold or silver atoms (typically 2–30 atoms) with physical sizes close to the Fermi wavelength of an electron (~0.5 nm for gold and silver) [34]. At such a small length scale, the density of states becomes discrete [35], giving metal clusters “molecular behaviors” and yielding fluorescence emission in the UV to near IR range [34]. The first organic synthesis produced metallic clusters with little fluorescence [36,37,38]. In early 2000’s Dickson’s group first reported the synthesis of highly fluorescent gold [39] and silver [24,40] nanoclusters in aqueous solution, opening up new opportunities for noble metal nanoclusters as biological labels. 

**Figure 1:**
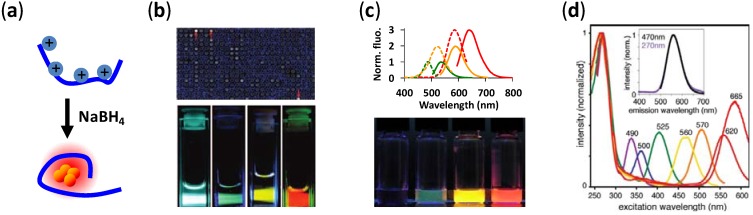
(**a**) Illustration depicting how silver nanoclusters are formed on single-stranded DNA. Silver ions preferentially attach to cytosines on the DNA strand. Addition of NaBH_4_ reduces these ions, leading to the formation of silver nanoclusters. The number of silver atoms in the cluster drawn here may not represent the real situation, as 10–20 atoms of silver could bind to a DNA strand [61,62,63,64]. (**b**) DNA microarrays proved to be a useful screening tool to find DNA sequences capable of nucleating Ag NCs of different emission colors. Reprinted with permission from [27]. Copyright (2008) American Chemical Society. (**c**) It is possible to switch the fluorescence of Ag NCs on and off [29,30] and tune their color [30,31] through interactions with nearby DNA sequences (called enhancers). Adapted with permission from [30]. (**d**) All DNA/Ag NCs have a common excitation peak between 260 and 270 nm, close to the absorption maxima of nucleobases. Through energy transfer from nucleobases, one can excite all DNA/Ag NCs with a single UV excitation source. Reprinted with permission from [53]. Copyright (2011) American Chemical Society.

**Figure 2:**
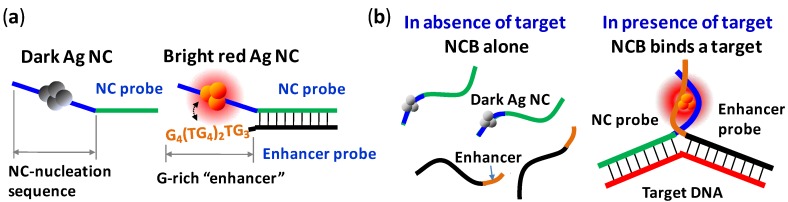
Schematic showing the guanine-proximity-induced fluorescence enhancement phenomenon on DNA/Ag NCs and its application in DNA detection—termed NanoCluster Beacons (NCBs). (**a**) Guanine proximity can dramatically increase the red fluorescence emission of DNA/Ag NCs. A non-emissive silver cluster is first prepared on a cytosine-rich NC-nucleation sequence. Once a guanine-rich enhancer is brought close to the silver cluster through hybridization, a strong red fluorescence emission is observed from the solution, with a bulk enhancement ratio greater than 500 fold. Reprinted with permission from [29]. Copyright (2010) AmericanChemical Society. (**b**) Representation of the NCB detection scheme. An NCB is composed of an NC probe (carrying a non-emissive Ag cluster) and an enhancer probe (having an enhancer sequence). NCB fluoresces upon binding with a DNA target. In the absence of target, NCB remains dark. Adapted with permission from [30].

Compared to gold nanoclusters, silver nanoclusters are brighter [34] and can be easily synthesized by using a number of ligands as stabilization agents (also called “encapsulation agents” or “templates”), including zeolites [41,42], PAMAM [40,43], PMMA [44], polyacrylate [45,46], poly(NIPAM-AA-HEA) microgels [47], sugar molecules [48], mercaptosuccinic acid [49], lipoic acid [50], thiol ligands [51], peptides [52] and DNA [24,26,28]. Among the different Ag NCs synthesized, ssDNA-templated silver nanoclusters (DNA/Ag NCs, Figure 1(a)) have become the center of research focus due to three key advantages. First, the fluorescence emission of DNA/Ag NCs can be tuned throughout the visible and near-IR range by simply programming the template sequences (Figure 1(b)). A complementary palette of DNA/Ag NC fluorophores has been produced [27,28]. Second, fluorescence of Ag NCs can be switched on and off [29,30] or color tuned [30,31] through interactions with a nearby DNA sequence (called an enhancer, see the next section) (Figure 1(c), Figure 2(a)). These emerging properties allow DNA/Ag NCs to be used not only as fluorescent tags but as sensors/indicators, leading to a variety of new biosensing opportunities, especially in DNA/RNA detection. Third, all DNA/Ag NCs share a similar UV excitation feature, regardless of the location of their visible excitation peaks (Figure 1(d)) [53]. In other words, it is possible to use a single UV source to excite all silver cluster species templated on DNA for multiplexed detection.

Other than the above advantages, DNA/Ag NCs have more benefits, including small size (as compared to semiconductor quantum dots) [25,27,54], low cellular toxicity [55,56,57], and are less prone to blinking on long (>1 ms) timescales [25,27,54]. DNA/Ag NCs can be brighter [25,54,58] and more photostable than organic dyes [25,54,59]. The synthesis can be done at room temperature via sequential mixing of four components: buffer, a cytosine-rich oligonucleotide, silver salt, and reducing reagent. Proper selection of buffer, buffer pH, and the reaction molar ratio enables the preferential formation of Ag nanoclusters (2–30 atoms) over larger plasmonic Ag nanoparticles. Readers are recommended to refer to the previous reports for further information on silver cluster preparation conditions [26,29,60]. In general, the synthesis starts with mixing micromolar DNA with 6–12 equivalents of silver nitrate (AgNO_3_) for 15 min in sodium phosphate buffer (pH 6.5 to 7.0), followed by adding 6–12 equivalents of sodium borohydride (NaBH_4_) to reduce the silver ions. The mixture is vortexed for one minute and left in the dark for 12 h [29].

While DNA/Ag NCs have demonstrated applications in single-molecule microscopy [25], biological imaging [59,65,66], molecular logic devices [67], metal ion sensing [68,69] and protein detection [70,71,72], a substantial advance (where silver nanoclusters were proved superior to organic dye or quantum dot alternatives) was made in DNA/RNA detection by the introduction of NanoCluster Beacons (NCBs) [29,30,31]. NCBs are new molecular probes that fluoresce upon hybridization with targets but do not use FRET as the fluorescence on/off switching mechanism, which is detailed below.

## 3. NanoCluster Beacons

We first discovered that DNA/Ag NCs could switch between bright and dark states by guanine proximity (Figure 2(a)), amounting to bulk fluorescence changes of more than 500 fold [29]. The fluorescence enhancement ratio was found to grow exponentially with an increasing number of guanine bases brought into proximity of Ag clusters. Based on the single-molecule imaging and fluorescence correlation spectroscopic results, the overall enhancement was caused by an increase in the number of silver clusters “turned on” due to guanine proximity. The fluorescence background of NCBs was attributed to the existence of sparse NCBs that fluoresced in absence of target. Charge transfer from guanines (the best electron donor among the four nucleobases [73]) to Ag clusters was initially thought to be responsible for the observed guanine-proximity-induced fluorescence turn-on phenomenon. Charge transfer-based fluorescence on/off switching was previously employed by Sauer’s group to create Smart Probes [74,75]. However, a guanine analog and an even stronger electron donor, 7-deazaguanine [75], was tested and found to fail at turning on the fluorescence of silver clusters, significantly weakening the charge transfer hypothesis as the physical phenomenon responsible for the red-enhanced fluorescence of these DNA-templated silver nanoclusters.

While the underlying mechanism for the guanine-proximity-induced fluorescence turn-on phenomenon is still under investigation, we have seen a great value in using this new signal transduction mechanism for biological or chemical sensing. We termed turn-on probes based on guanine-induced fluorescence enhancement NanoCluster Beacons (hereafter, denoted as NCBs) [29]. As illustrated in Figure 2(b), the first NCB design consists of two linear probes, one bearing a non-emissive silver nanocluster (*i.e.*, the NC probe) and the other having a guanine-rich tail (*i.e.*, the enhancer probe). The two probes are designed to bind in juxtaposition to a target DNA, allowing the enhancer (*i.e.*, the guanine-rich tail) to interact with the Ag cluster, converting the cluster from a non-emissive to a highly fluorescent state. Fluorescence occurs only when a specific nucleic acid target is present in the sample.

Compared to FRET-based turn-on probes such as molecular beacons [13], NCBs only require a one-step preparation process (*i.e.*, Ag cluster nucleation process on the NC probe) and no purification steps (the excess precursors, Ag^+^ and BH_4_^−^, are essentially non-fluorescent). NCBs are therefore inexpensive, easy to prepare, and compatible with commercial DNA synthesizers (fluorescence enhancement caused by intrinsic nucleobases). As mentioned in the Introduction, the level of quenching in a FRET probe is never 100% efficient, therefore limiting the single-to-background (S/B) ratio that a FRET probe can achieve in biosensing. On the other hand, not relying on FRET as on/off switching mechanism, we have demonstrated NCB detection of an influenza target with an S/B ratio five times better than that of a conventional molecular beacon [29]. While our original work used NCBs for nucleic acid detection, similar methods have since been developed to detect other biomolecules of interest [71,76]. Moreover, since this initial discovery, we have turned NCBs into multi-color probes using two strategies: (1) by employing the same enhancer sequence but different NC-nucleation sequences [30] or (2) by employing the same NC-nucleation sequence but different enhancer sequences [29,30]. This important feature, having multiple “turn-on colors” from the same origin, is not commonly shared by organic dyes or semiconductor quantum dots, opening opportunities for NCBs in multiplexed assays.

**Figure 3:**
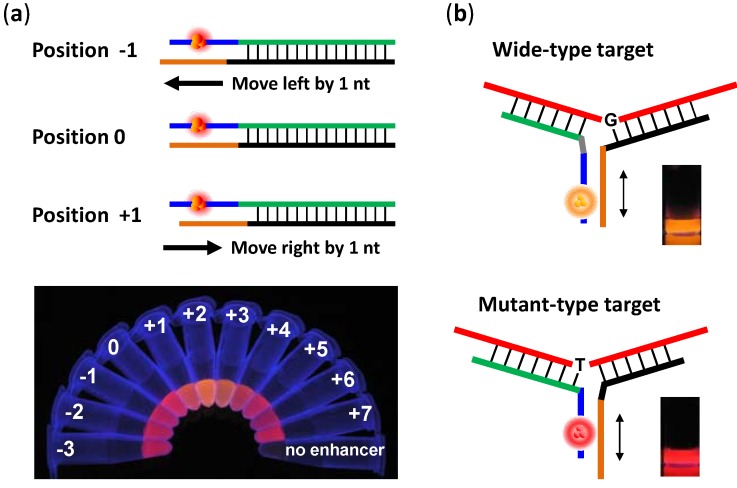
(**a**) The turn-on color of NanoCluster Beacons can be tuned by repositioning the enhancer sequence with respect to the NC-nucleation sequence. Schematic shows the relative positions between the enhancer sequence (lower strand, orange line) and the NC-nucleation sequence (upper strand, blue line). The photo shows the associated emission colors under UV excitation. (**b**) Chameleon NanoCluster Beacons, which take advantage of the repositioning-induced-color-tuning phenomenon, light up into different colors upon binding with distinct SNP targets. Adapted with permission from [31]. Copyright (2012) AmericanChemical Society.

Lately we demonstrated an entirely new phenomenon—the turn-on color of the silver cluster can change substantially depending upon its position relative to an enhancer sequence [31] (Figure 3(a)). Based on this new discovery, we have transformed NCBs from a “turn-on” probe into a “color-switching/turn-on” probe, termed “chameleon” NanoCluster Beacons, cNCBs [31] (Figure 3(b)). The key benefit of this color-switching version of NCBs lies in their capability for a two-dimensional analysis—the fluorescence intensity can be used to quantify the amount of DNA target, whereas fluorescence color can be used to identify the single-nucleotide polymorphisms (SNPs) on the target. Again, this feature is not shared by existing SNP probes [77,78,79], where events without a target cannot be easily differentiated from events with a mismatched target (both give a low signal). Owing to the unique features of NCBs and cNCBs, we envision that NCBs will find more sensing applications in the near future. 

## 4. Other DNA/Ag NC-Based DNA/RNA Detection Methods

### 4.1. Detection of DNA Targets

**Figure 4:**
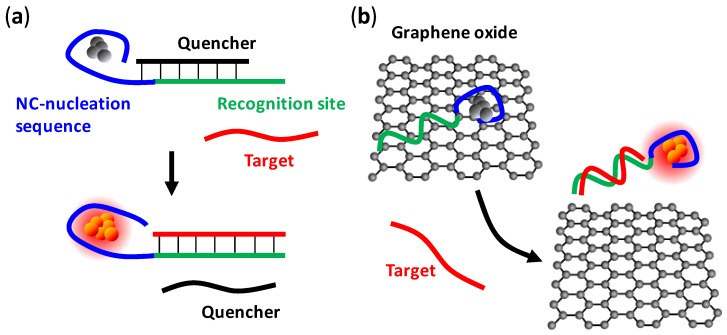
(**a**) Representation of a quencher-mediated turn-on probe. After the quencher (black) is displaced by the target (red), fluorescence is enhanced by a proportional increase in the number of emissive clusters in the sample [80]. (**b**) Representation of a DNA detection assay using DNA/Ag NCs and graphene oxide (GO) nanohybrids. DNA/Ag NCs serves as a reporter while GO is a quencher. Silver clusters templated on the ssDNA are initially quenched by the GO. Once the adsorbed probe hybridizes with a target, the duplex is released from the GO and the fluorescence of the silver cluster is restored [83].

Petty’s group reported a turn-on nanocluster probe that has silver clusters initially quenched through hybridization with a quencher strand. As shown in Figure 4(a), the DNA target binds to the NC probe through a competitive process called toehold displacement [80]. While eliminating the need of a guanine-rich enhancer for turning on the fluorescence, this method gives only 2 to 3 fold of fluorescence enhancement upon target binding. Petty and Dickson have previously characterized the near-infrared-emitting silver cluster species (emission maxima around 770 nm) templated within their NC-nucleation sequence, 5′-C_3_AC_3_AC_3_GC_3_A. Using inductively coupled plasma atomic emission spectroscopy, a stoichiometry of 10 silver atoms per oligonucleotide was determined [63]. Similar to our results, Petty also found that fluorescence enhances through a proportional increase in the number of emissive clusters [80]. As for their turn-on mechanisms, they believe quencher invasion into the 3' region of the NC-nucleation sequence might have driven the silver cluster to a binding site having a different electronic environment, leading to the observed quenching effect. The key advantage of this method lies in the emitter’s near-infrared electronic transition, which enables DNA detection in serum-containing buffers (whose endogenous background fluorescence is low in the near-infrared spectral region). However, it is not clear whether or not the detection scheme shown in Figure 4(a) can be applied to longer DNA targets. 

Petty’s turn-on probe has evolved to a new form where the quencher strand is no longer needed [64] (similar results also achieved by Chang’s group [81]). The new probe relies on the capability of silver cluster (~11 silver atoms) to condense the NC probe for fluorescence quenching (*i.e.*, creating an environment favoring the dark state of the silver clusters). Target hybridization transforms the cluster environment and therefore restores the fluorescence of silver clusters. Interestingly, similar to the self-dimer formation reported by Yang and Vosch in their turn-off probe design [82], Petty also found the probe-target hybrids form dimers, hosting twice the amount of silver atoms in a dimer [64]. It is suggested that the dimer formation of the DNA/Ag NC probes is responsible for the creation and stabilization of highly emissive silver clusters [82], but the underlying mechanism is currently unclear. 

Graphene oxide (GO) has emerged as an effective universal quencher that can initially switch off the fluorescence of reporters in turn-on detection [83,84]. Single-stranded DNA (ssDNA) can be stably adsorbed by the GO through π-stacking interactions between the ring structures in the nucleobases and the hexagonal cells of the graphene. As shown in Figure 4(b), once near the GO, the “reporters” labeled on the ssDNA are quenched by the GO. In the presence of DNA targets, the adsorbed probes hybridize with the targets, forming rigid duplexes which are then released from the GO due to conformational changes. This releasing mechanism reverses the quenching effect and restores the fluorescence. Ren’s group recently used DNA/Ag NCs as reporters in a GO-based sensor for multiplexed detection of DNA [85], demonstrating the compatibility between DNA/Ag NCs and fullerene. 

### 4.2. Detection of Single-Nucleotide Polymorphisms

**Figure 5:**
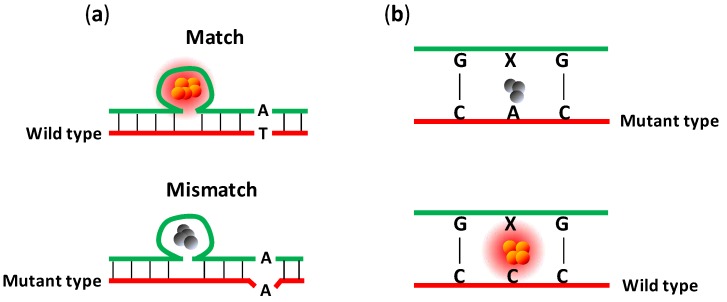
(**a**) Representation of hybridized DNA structures with a six-cytosine loop as nucleation site for Ag NC synthesis and their use in SNP detection. No fluorescence was observed for the mutant-type duplex after the nucleation process. However, the wild-type duplex produced strong yellow fluorescence emission [32]. (**b**) Representation of DNA abasic site-directed nucleation of emissive Ag NCs for selective nucleobase recognition [86]. When a cytosine was placed opposite to the abasic site, strong fluorescence emission was seen. On the other hand, when adenine, thymine, or guanine was placed opposite the abasic site, weak fluorescence was observed.

Several methods (in addition to cNCBs [31]) have been developed to use DNA/Ag NCs to detect single-nucleotide polymorphisms (SNPs). In work done by Wang’s group, identification of the SNPs responsible for sickle cell anemia was achieved using a probe with a six-cytosine, NC-nucleation loop (Figure 5(a)) [32]. The probe was first hybridized with the wild-type and the mutant-type targets (T to A substitution), forming two different duplexes. After performing NC-nucleation process on these duplexes, almost no fluorescence was observed from the mutant-type duplex, whereas the wild-type duplex produced a strong yellow fluorescence emission. However, unlike cNCBs, this method differentiates single-nucleotide variants by the level of fluorescence intensity. It requires nanocluster synthesis process to take place after probe/target hybridization is completed, leading to a longer assay time. Moreover, it is not clear whether or not such a method can be generally applied to a wide variety of target sequences and still obtain similar discrimination results.

Shao’s group demonstrated that an abasic site in the DNA duplex can serve as a nucleation site for the synthesis of emissive Ag NCs and they used this property to differentiate single-nucleotide variants [86]. As shown in Figure 5(b), when a cytosine was placed opposite to the abasic site (denoted as X, a tetrahydrofuran residual), strong fluorescence emission was seen after the NC nucleation process. On the other hand, when adenine, thymine, or guanine was placed opposite to the abasic site, weak fluorescence was observed. In this fashion, Shao’s group was able to differentiate a cytosine from other SNP variants. However, this method can only differentiate C → A, C → G, and C → T mutations but not any other type of substitutions.

### 4.3. Detection of RNA Targets

DNA/Ag NCs also offer the potential for sensitive and quantitative detection of RNA, in particular the microRNA. MicroRNA’s (miRNA) are small non-coding ribonucleic acids that can regulate the genes associated with cancer, neurological diseases and viral infections. Ye’s group demonstrated miRNA detection using an isothermal amplification method, where DNA/Ag NCs were used as fluorescent reporters for the amplicons [87]. In their approach, the amplicons are single-stranded DNA that can nucleate the growth of highly emissive Ag NCs.

Instead of using the fluorescence emission of DNA/Ag NCs to quantify miRNA concentrations, other groups have used fluorescence quenching of DNA/Ag NCs as the basis of detection. In the work of Yang and Vosch, a DNA/Ag NC probe was developed whose fluorescence was quenched when it hybridized to a complementary miRNA target [82,88]. Fluorescence quenching was shown to follow a linear Stern-Volmer relationship over a range of target concentrations (from 20 nM to 1.5 μM), enabling quantitative detection of target miRNA. 

In addition to fluorescence-based measurements, DNA/Ag NCs have also been exploited for the sensitive detection of miRNA by electrochemical methods. For example, Zhang’s group developed a simple miRNA sensor using DNA/Ag NCs to catalyze the reduction of H_2_O_2_ [89]. In their method, thiol-functionalized DNA hairpin probes were immobilized on the surface of a gold electrode, with these hairpin probes having binding sites for both miRNA targets and NC-nucleation probes. Upon sequential binding of miRNA and NC-nucleation probes (which carry Ag NCs), a large number of Ag NCs were brought close to the electrode’s surface, enabling catalytic reduction of H_2_O_2_. The detected current was found directly proportional to the logarithm of the miRNA target concentration.

## 5. Future Prospects

While DNA/Ag NCs have been successfully exploited for bio-labeling, imaging, and DNA/RNA detection, we feel there is still tremendous potential for growth in the use of DNA-templated silver nanoclusters. For example, they may see use in the labeling of mRNA in live cells for 2D [90] or potentially 3D [91,92,93,94] mRNA tracking studies. Another area of potential growth lies in the development of a new class NanoCluster Beacons that could discriminate four SNP types co-existing in a single reaction, while the current state-of-the-art can only discriminate three SNP types at once [31]. 

At present, a number of highly sequence-dependent DNA/Ag NCs with superior fluorescence emission and multi-color switching properties have been synthesized and optimized, which could see increased use in multiplexed detection. For example, over a decade ago Nie’s group demonstrated the use of semiconductor quantum dots to encode polymer beads of different emission colors and intensities [95]. The smaller size of DNA/Ag NCs and their non-toxic core material may make these superior fluorophores a better choice in creating encoded microspheres.
